# The effect of hyperuricemia and its interaction with hypertension towards chronic kidney disease in patients with type 2 diabetes: evidence from a cross- sectional study in Eastern China

**DOI:** 10.3389/fendo.2024.1415459

**Published:** 2024-07-29

**Authors:** Xiang-yu Chen, Feng Lu, Jie Zhang, Chun-xiao Xu, Xiao-fu Du, Ming-bin Liang, Li-jin Chen, Jie-ming Zhong

**Affiliations:** Department of Non-Communicable Disease Control and Prevention, Zhejiang Provincial Center for Disease Control and Prevention, Hangzhou, China

**Keywords:** hyperuricemia, hypertension, interaction, chronic kidney disease, diabetes mellitus

## Abstract

**Objectives:**

This study aimed to explore the synergistic interaction effect between hyperuricemia and hypertension towards chronic kidney disease in patients with type 2 diabetes.

**Methods:**

This research originates from a cross-sectional study performed in Zhejiang Province, Eastern China, between March and November 2018. The correlation between serum uric acid levels and the risk of chronic kidney disease was assessed using a restricted cubic spline model. An unconditional multivariable logistic regression model, along with an interaction table, was utilized to explore the potential interaction effect of hyperuricemia and hypertension towards chronic kidney disease.

**Results:**

1,756 patients with type 2 diabetes were included in this study, the prevalence of chronic kidney disease (CKD) was 27.62% in this population. A U-shaped non-linear pattern emerged correlating serum uric acid (SUA) levels and CKD risk, indicating that both low and high SUA levels were linked to an increased CKD risk. This risk achieved its lowest point (nadir) at SUA approximately equals to 285μmol/L (p for trend <0.05). Once adjustments for age, gender, education level, abnormal fasting plasma glucose (FPG), abnormal hemoglobin A1c (HbA1c), abnormal total cholesterol (TC), abnormal high-density lipoprotein cholesterol (HDL-C), alcohol consumption and duration of diabetes were factored in, it was found that patients with both hyperuricemia and hypertension demonstrated a 5.42-fold (95% CI: 3.72–7.90) increased CKD risk compared to the reference group. The additive interaction between hyperuricemia and hypertension was statistically significant, as manifested by the following values: a relative excess risk due to interaction (RERI) of 2.57 (95% CI: 0.71–4.71), an attributable proportion due to interaction (AP) of 0.47 (95% CI: 0.14–0.64), and a synergy index (SI) of 2.39 (95% CI: 1.24–4.58). In contrast, there was no significant interaction effect in multiplicative scale.

**Conclusion:**

Hyperuricemia and hypertension may contribute additively to CKD, beyond their isolated impacts. Evaluating the risk of CKD in type 2 diabetes patients necessitates considering this potential interaction.

## Introduction

In 2019, the number of Chinese adults with diabetes was 111.6 million, and it is expected to rise to 147 million by 2045 (1). This trend poses a significant public health concern globally (2). Chronic kidney disease (CKD), defined as a range of heterogeneous diseases manifesting chronic renal structural and functional abnormalities, the incidence, disability, and mortality rates associated with CKD have shown dramatic increases, making it a major global health challenge (3–5).Notably, CKD is frequently seen in individuals with type 2 diabetes (T2DM), ranking it as a leading cause of end-stage renal disease (ESRD) (6). Consequently, devising preventative measures for CKD presents a complex challenge. In response to this urgent need, it is crucial to carefully investigate the risk factors linked to CKD, assessing their potential interactions, to address this significant unfulfilled health need.

It is noted that hypertension(HTN) and T2DM frequently coexist, with approximately 70% of the T2DM patients also having HTN (7). Hypertension is also critically involved in the progression of CKD. The underlying mechanisms through which HTN precipitates kidney dysfunction encompass elevated intraglomerular pressure, inflammation, oxidative stress, and endothelial dysfunction, etc.Hyperuricemia (HUA), a metabolic abnormality syndrome arising from perturbed purine metabolism, demonstrates multiple mechanisms that detrimentally impact renal function (8).. Notably, excessive uric acid (UA) accumulation leads to deteriorated renal performance, which then exacerbates the UA build-up within the system, engendering a detrimental feedback loop of increasing UA deposition in the kidneys. A previous study highlighted the synergistic effect of HUA and HTN on the reduction of the estimated glomerular filtration rate (eGFR) in the general Chinese population (9). Nonetheless, it remains uninvestigated whether these two factors act synergistically towards CKD in patients with T2DM.

The aim of this study is to investigate the characteristics of CKD in patients with T2DM. We will examine the effects of HUA and its interaction effect with HTN towards CKD, employing data from the Zhejiang Provincial Diabetic Complications Study. The findings will provide insightful reference for strategies in preventing and managing CKD among this T2DM population.

## Methods

### Study design and population

This research endeavor, spanning March to November 2018, was an integral component of the China National Diabetic Complications Study. The investigation aimed to discern the prevalence and risk factors related to diabetic complications among individuals with T2DM in Zhejiang Province, Eastern China (10). Comprehensive details pertaining to the design, methods, and participants of the study are available in additional sources (11). In summary, the study focused on eligible individuals with T2DM, resident at the survey sites for a minimum of six months within the preceding year. The participant inclusion criteria stipulated that they must be at least 18 years of age, cognitively competent, non-pregnant, and not bedridden.

This study employed a multi-stage random sampling approach. Initially, two districts and two counties from Zhejiang Province were arbitrarily selected. Then, from each of these districts and counties, four streets or towns were randomly chosen. The final stage involved randomly selecting 120 T2DM patients, registered with the local healthcare system, from each street or town. The selection was stratified on the basis of gender and age, culminating in a total of 1,920 eligible participants. The study incorporated a thorough physical examination, fasting blood sampling, and a detailed questionnaire survey, all of which were administered face-to-face to every participant (10)..

Prior to data collection, the present research project received approval from the ethics committee (Approval No: 2018–010) on January 13, 2018, and was registered in the Chinese Clinical Trial Registry (ChiCTR1800014432). Each participant in the study provided informed consent in a written format (10). The study was conducted strictly in accordance with the Declaration of Helsinki.

### Data collection and measurements

The questionnaire survey was conducted by trained staff from the local Center for Disease Control and Prevention (CDC) and local primary healthcare centers. They collected information on participant demographics and health-related behaviors through direct, orally administered questionnaires. Physical examinations of participants, which involved the measurement of body height, weight, waist circumference, and blood pressure, were carried out at the primary healthcare centers by skilled staff. The participants’ height was measured to a precision of 0.1 centimeters (cm) using a TZG height gauge(Suhong,China), and body weight was gauged to a precision of 0.1 kilograms (kg) using a HD-390 scale(TANITA, Japan). Blood pressure was recorded thrice with a 1-minute interval between measurements, employing HBP-1300 electronic blood pressure monitor(OMRON, Japan). This study assessed multiple indicators, sourced from both fasting venous blood samples and random urinary samples from the participants. These indicators included fasting plasma glucose (FPG), glycosylated hemoglobin (HbA1c), serum uric acid (SUA), triglycerides (TG), total cholesterol (TC), low-density lipoprotein cholesterol (LDL-C), high-density lipoprotein cholesterol (HDL-C), serum creatinine (Scr), urinary albumin (UAlb), and urinary creatinine (Ucr). Enzyme-based methods were used to measure lipids (TC, TG, HDL-C, LDL-C), SUA, Scr, and Ucr, utilizing a Roche cobas c701 automated analyser(Roche, Switzerland). FPG was determined via hexokinase assay, and HbA1c was gauged using high-performance liquid chromatography on a Hemoglobin Analyzer(Bio-Rad, USA). Finally, UAlb was measured through the immunoturbidimetry method using a Roche cobas c701 automated analyser(Roche, Switzerland).

### Definition of the variables

The outcome variable for this study was CKD. CKD was defined as cases having either an eGFR<60 ml/min per 1.73 m^2^ or a urinary albumin-to-creatinine ratio(UACR) ≥30mg/g (12). eGFR was calculated using CKD-EPI formula (13). Hypertension was defined according to the “Chinese hypertension guidelines”: systolic blood pressure(SBP) ≥140mmHg and/or a diastolic blood pressure(DBP) ≥90mmHg in conjunction with self-reported diagnosis of HTN (14). Hyperuricemia was defined according to the “Chinese multidisciplinary expert consensus on the diagnosis and treatment of hyperuricemia and related diseases”: SUA levels exceeding 420 μmol/L for both sexes (15).Age was categorized into three distinct classes: young adults, encompassing those 18 to 44 years; middle-aged adults, 45 to 59 years; and senior adults, 60 or above. Educational status was classified in three tiers: secondary school or lower, senior high school, and college or above. Participants were designated as dwelling in either urban or rural areas based on their place of residence. Current cigarette smoking status was confirmed by daily or occasional cigarette usage, while alcohol drinking was identified for those a consummation of alcohol within the preceding thirty days. FPG and HbA1c were deemed abnormal for readings ≥7.0 mmol/L and≥7.0% respectively. Duration of diabetes was defined as follows: 5 years or less, 6–10 years, 11–15 years, more than 15 years.

### Statistical analysis

Continuous data were expressed as mean ± standard deviation or median (interquartile range) with differences between groups analyzed using t-tests or Wilcoxon rank-sum tests. Categorical data were presented as frequency (percentage) and chi-square test (χ^2^ test) was used to compare differences between groups. To evaluate the correlation between SUA and CKD risk, a restricted cubic spline model was employed. An unconditional multivariate logistic regression model was also used to identify CKD-related influencing factors, with the backward elimination method used for adjusting confounders. Additionally, the interaction impact between HUA and HTN was examined on both multiplicative and additive scales (16, 17).The computation of the Relative Excess Risk due to Interaction (RERI), Attributable Proportion due to Interaction (AP), and Synergy Index (SI) were undertaken. The 95% confidence intervals (CIs) for interaction indicators were calculated based on T. Anderson’s table (18). A RERI value of 0 signifies the absence of additive interaction whereas a RERI above 0 signifies a positive additive interaction (19). The existence of an interaction is excluded if the 95% CI, for either the AP or RERI, contains 0 or if the 95% CI for the SI includes 1.In relation to the multiplicative interaction, the term HUA*HTN was incorporated into the multivariate logistic regression models as an interaction variable (9). The significance and intensity of the multiplicative interaction were evaluated using the OR associated with the interaction term. The statistical significance level (α) was set at 0.05. SAS software (version 9.4) was used for data analysis.

## Results

### Basic characteristics of the cases

The current study involved analysis of data from 1,756 participants who had provided complete research information. The baseline characteristics of the cases are summarized in **Table 1**. The distribution of gender within the group indicated that 876 participants, or 49.89%, were male. The average age of participants was 57.23 ± 10.15 years, with a mean BMI of 24.76 ± 3.43 kg/m^2^. The distribution of diabetes duration among the study cases is as follows: 836 (47.61%) cases with 5 years or less, 491 (27.96%) cases with 6–10 years, 236 (13.44%) cases with 11–15 years, and 491 (10.99%) cases with more than 15 years. Among the study cases, 263 (14.98%) cases did not receive medication, 1,207 (68.74%) cases received only anti-hyperglycemic medications, 87 (4.95%) cases received only insulin treatment, and 199 (11.33%) cases received both anti-hyperglycemic medications and insulin treatment. CKD was present in 485 (27.62%) cases, in which 70 (14.43%) cases with a low eGFR(eGFR<60 ml/min per 1.73 m^2^), 352 (72.58%) with a high UACR (UACR≥30mg/g) and 63 (12.99%) with both. HTN was present in 1,133 (64.52%) while HUA was present in 292 (16.63%).

**Table 1:** Basic characteristics of the cases classified by CKD ( n=1,756).

Characteristics	Overall(n=1,756)	Group without CKD(n=1,271)	Group with CKD(n=485)	t/χ^2^/z	p
Age(years) [means±SD]	57.23±10.15	56.52±9.92	59.09±10.54	-4.77 ^a^	<0.001
Gender,n(%)				0.048^b^	0.827
Male	876(49.89)	632(49.72)	244(50.31)		
Female	880(50.11)	639(50.28)	241(49.69)		
Educational level,n(%)				8.12 ^b^	0.017
Secondary school and lower	1,541(87.76)	1130(88.90)	411(84.74)		
Senior high school	171(9.74)	108 (8.50)	63 (12.99)		
College or above	44(2.50)	33(2.60)	11(2.27)		
Residence,n(%)				1.30 ^b^	0.255
Rural	875(49.83)	644(50.67)	231 (47.63)		
Urban	881(50.17)	627(49.33)	254(52.37)		
BMI (kg/m^2^) [means±SD]	24.76±3.43	24.51±3.35	25.43±3.56	-5.06 ^a^	<0.001
SBP(mmHg) [means±SD]	136.45±18.71	133.54±17.37	144.09±19.92	-10.27 ^a^	<0.001
DBP(mmHg) [means±SD]	78.39±10.68	77.57±10.20	80.55±11.60	-4.98 ^a^	<0.001
HTN,n(%)	1133(64.52)	739(58.14)	394(81.24)	81.79^b^	<0.001
TG (mmol/L) [median(IQR)]	1.60(1.12-2.42)	1.51(1.06-2.26)	1.87(1.30-2.94)	47.47^c^	<0.001
TC (mmol/L) [means±SD]	4.65±1.07	4.61±0.97	4.78±1.29	-2.64 ^a^	0.009
HDL-C (mmol/L) [means±SD]	1.25±0.36	1.28±0.35	1.18±0.37	4.96 ^a^	<0.001
LDL-C (mmol/L) [means±SD]	2.73±0.90	2.75±0.85	2.70±1.02	0.98 ^a^	0.325
FPG (mmol/L) [means±SD]	7.94±2.58	7.72±2.31	8.54±3.11	-5.32 ^a^	<0.001
HbA1c(%) [means±SD]	7.27±1.49	7.14±1.40	7.61±1.65	-5.43 ^a^	<0.001
SUA (mmol/L) [means±SD]	334.65±94.44	325.89±85.50	357.60±111.48	-5.66 ^a^	<0.001
HUA,n(%)	292(16.63)	171(13.45)	121(24.95)	33.46^b^	<0.001
Scr(μmol/L) [median(IQR)]	71.00 (59.00-85.00)	69.00 (59.00-81.00)	80.00 (63.00-99.00)	82.03^c^	<0.001
UAlb(mg/L) [median(IQR)]	17.30 (6.90-43.40)	11.10 (4.95-22.05)	87.10 (41.95-228.00)	705.80^c^	<0.001
Ucr(μmol/L) [median(IQR)]	12735.00 (8799.00-18085.50)	13200.00 (9380.50-18630.50)	11510.50 (7689.00-16537.00)	20.04^c^	<0.001
Duration of diabetes(years),n(%)				23.52^b^	<0.001
<=5	836(47.61)	635(49.96)	201(41.44)		
6-10	491(27.96)	364(28.64)	127(26.19)		
11-15	236(13.44)	148(11.64)	88(18.14)		
>15	193(10.99)	124(9.76)	69(14.23)		
Therapies of diabetes,n(%)				35.57^b^	<0.001
No medication	263(14.98)	201(15.81)	62(12.78)		
Anti-hyperglycemic drugs only	1207(68.74)	904(71.13)	303(62.47)		
Insulin only	87(4.95)	52(4.09)	35(7.22)		
Anti-hyperglycemic drugs and insulin	199(11.33)	114(8.97)	85(17.53)		

^a^Student’s t-test; ^b^Chi-square test; ^c^Wilcoxon rank-sum test.

Abbreviations: CKD, chronic kidney disease; BMI,body mass index; SBP, systolic blood pressure; DBP,diastolic blood pressure; HTN, hypertension; TG,triglycerides; TC, total cholesterol; HDL-C, high density lipoprotein-cholesterol; LDL-C, low density lipoprotein-cholesterol; FPG, fasting plasma glucose; SUA, serum uric acid; HUA, hyperuricemia; Scr, serum creatinine; UAlb,urinary albumin ;Ucr, urinary creatinine.

Multivariable analysis on influencing factors of CKD in patients with T2DM **Table 2** presents the multivariable logistic regression results pertaining to the influencing factors of CKD. The results showed that aside from HTN and HUA, age, educational level, FPG abnormal, HbA1c abnormal, TC abnormal, HDL-C abnormal and duration of diabetes were significantly associated with an elevated risk of CKD. After controlling for these variables, the risk of CKD was found to be 1.93 times higher in T2DM patients with HUA, compared to those without HUA (95%CI: 1.45–2.58). Similarly, the risk of CKD was 2.80 times higher in T2DM patients with HTN compared to those without HTN (95%CI: 2.13–3.70).

**Table 2:** Multivariable analysis on influencing factors of CKD (n=1,756).

Variables		Group without CKD (n=1,271)	Group with CKD (n=485)	OR(95%CI)	p
Gender, n(%)	Female	639 (50.28)	241(49.69)		
	Male	632 (49.72)	244 (50.31)	0.96(0.75-1.24)	0.766
Age (Years), n(%)	18-44	195 (15.34)	68 (14.02)		
	45-59	601 (47.29)	166 (34.23)	0.65 (0.45-0.94)	0.022
	≥60	475 (37.37)	251 (51.75)	1.21 (0.83-1.76)	0.321
Educational level, n(%)	Secondary school and lower	1130 (88.91)	411 (84.74)		
	Senior high school	108 (8.50)	63 (12.99)	2.10(1.45-3.04)	<0.001
	College or above	33 (2.60)	11 (2.27)	1.17 (0.54-2.53)	0.686
HUA, n(%)	No	1100 (86.55)	364 (75.05)		
	Yes	171 (13.45)	121 (24.95)	1.93 (1.45-2.58)	<0.001
HTN, n(%)	No	532 (41.86)	91 (18.76)		
	Yes	739 (58.14)	394 (81.24)	2.80 (2.13-3.70)	<0.001
FPG abnormal, n(%)	No	595 (46.81)	172 (35.46)		
	Yes	676 (53.19)	313 (64.54)	1.39 (1.07-1.81)	0.015
HbA1c abnormal, n(%)	No	691 (54.37)	206 (42.47)		
	Yes	580 (45.63)	279 (57.53)	1.42 (1.09-1.85)	0.009
TC abnormal, n(%)	No	1203 (94.65)	433 (89.28)		
	Yes	68(5.35)	52 (10.72)	2.08 (1.38-3.15)	<0.001
HDL-C abnormal, n(%)	No	949 (74.67)	301 (62.06)		
	Yes	322 (25.33)	184 (37.94)	1.57 (1.23-2.00)	<0.001
Alcohol drinking, n(%)	No	785 (61.76)	325(67.01)		
	Yes	486 (38.24)	160(32.99)	0.67 (0.52-0.87)	0.003
Duration of diabetes(years), n(%)	<=5	635(49.96)	201(41.44)		
	6-10	364(28.64)	127(26.19)	1.11 (0.84-1.47)	0.447
	11-15	148(11.64)	88(18.14)	1.69 (1.21-2.36)	0.002
	>15	124(9.76)	69(14.23)	1.59 (1.11-2.30)	0.012

CKD, chronic kidney disease; OR, odds ratio; CI, confidence interval; HUA, hyperuricemia; HTN, hypertension; FPG, fasting plasma glucose; TC, total cholesterol; HDL-C, high density lipoprotein-cholesterol.

### The association between CKD and SUA

**Figure 1:**
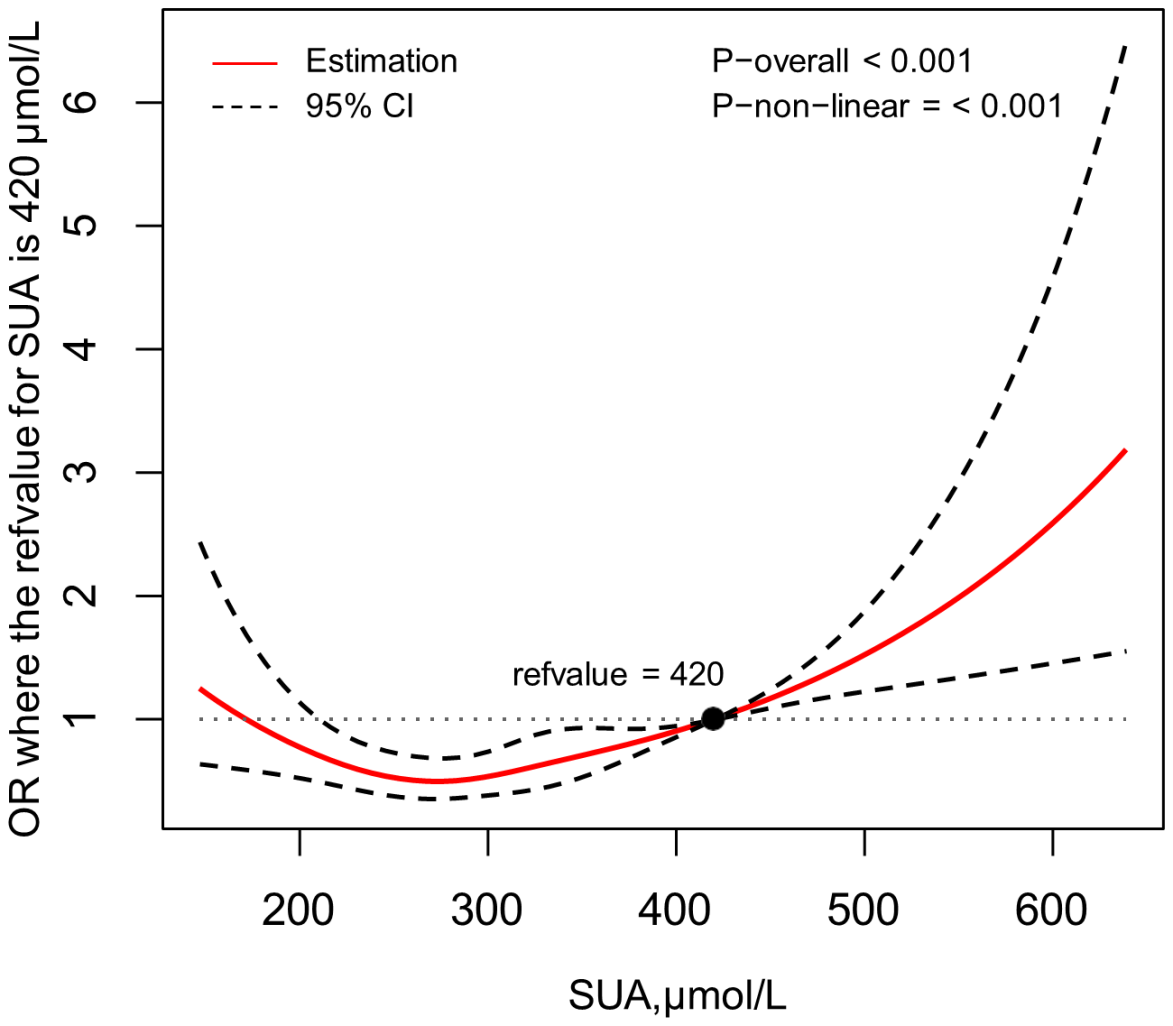
Association between SUA and the risk of CKD, allowing for nonlinear effects, with 95%CI. The model shows ORs compared with SUA=420μmol/L, adjusting for age, gender, educational level, HTN, FPG abnormal, HbA1c abnormal, TC abnormal, HDL-C abnormal, alcohol drinking and duration of diabetes. SUA, serum uric acid; CKD, chronic kidney disease; CI, confidence interval; OR, odds ratio; HTN, hypertension; FPG, fasting plasma glucose; TC, total cholesterol; HDL-C, high density lipoprotein-cholesterol.

In **Figure 1**, a restricted spline was utilized to depict the correlation between SUA levels and the risk of CKD, with the reference value (OR=1.00) for SUA set at 420μmol/L. This figure showed that after adjusted for age, gender, educational level, HTN, FPG abnormal, HbA1c abnormal, TC abnormal, HDL-C abnormal, alcohol drinking and duration of diabetes, there was a non-linear relationship between SUA levels and CKD risk (p for non-linearity < 0.05). The graph indicates that CKD risk diminishes as SUA levels rise up to a point of approximately SUA=285μmol/L, after which the risk steadily increases. As the cut-off value of HUA is still debated and probably a lower cut-off value of HUA may potentially contribute to CKD progression (20), therefore, we have also conducted this analysis on the HUA cut-off at a value of 5.6 mg/dL (336μmol/L). Setting the reference value (OR=1.00) for SUA at 336μmol/L also revealed this similar U-shaped relationship (**Supplementary Figure S1**).

### The interaction analysis between HUA and HTN on the risk of CKD

In order to evaluate the interaction effect of HUA and HTN on CKD, participants were categorized into four groups (non-HUA & non-HTN, non-HUA & HTN, HUA & non-HTN and HUA & HTN; **Table 3**). In the crude model, the HUA & HTN group exhibited a 5.33-fold risk (95% CI: 3.76–7.54) compared to the non-HUA & non-HTN group, a markedly higher risk than both the non-HUA & HTN group and the HUA & non-HTN group. With adjustments for age and gender, the risk for the HUA and HTN group lowered slightly decreased to 5.19 (95% CI: 3.63–7.43). After full adjustment, the resultant estimate was 5.42 (95% CI: 3.72–7.90), which still remained considerably higher than that of the non-HUA & HTN group (2.58, 95% CI: 1.93–3.45) and the HUA & non-HTN group (1.28, 95% CI: 0.61–2.69).

**Table 3:** Multivariate logistic regression models of the joint effect of HUA and HTN on CKD(n=1,756).

Models	Variables		OR(95%CI)	ORs(95%CI) of HTN within the strata of HUA	ORs(95%CI) of HUA within the strata of HTN
	HUA	HTN			
Crude	No	No	1.00(ref)		
		Yes	2.77 (2.10-3.64)	2.77 (2.10-3.64)	
	Yes	No	1.30 (0.63-2.69)		1.30 (0.63-2.69)
		Yes	5.33 (3.76-7.54)	4.08 (1.97-8.48)	1.93 (1.44-2.58)
Model 1	No	No	1.00(ref)		
		Yes	2.61 (1.97-3.46)	2.61 (1.97-3.46)	
	Yes	No	1.35 (0.65-2.80)		1.35 (0.65-2.80)
		Yes	5.19 (3.63-7.43)	3.84 (1.85-8.01)	1.99 (1.48-2.68)
Model 2	No	No	1.00(ref)		
		Yes	2.58 (1.93-3.45)	2.58 (1.93-3.45)	
	Yes	No	1.28 (0.61-2.69)		1.28 (0.61-2.69)
		Yes	5.42 (3.72-7.90)	4.25 (2.00-9.01)	2.11 (1.54-2.88)

HUA, hyperuricemia; HTN, hypertension; CKD, chronic kidney disease; OR, odds ratio; CI, confidence interval.

Crude: unadjusted any covariate; Model 1: adjusted for age, gender; Model 2: adjusted for age, gender, educational level, FPG abnormal, HbA1c abnormal, TC abnormal, HDL-C abnormal and alcohol drinking, duration of diabetes.

We undertook an interaction effect analysis to determine whether HUA and HTN display a synergistic interaction towards CKD, as detailed in **Table 4**. On the additive scale, three interaction indicators (RERI, AP and SI) showed significant and positive associations in the preliminary model. After accounting for age and sex, the values of these indicators remained relatively stable. Following full adjustment, these values demonstrated minor increases. In the fully adjusted model, the RERI, AP, and SI were recorded as 2.57 (95% CI: 0.71–4.71), 0.47 (95% CI: 0.14–0.64), and 2.39 (95% CI: 1.24–4.58), respectively. The RERI suggested a 2.57-fold risk out of a total 5.42-fold risk associated with the synergistic interaction, whilst the AP demonstrated that 47% of the total CKD risk could be attributed to the HUA and HTN interaction. However, on the multiplicative scale, no significant interaction effects on CKD were discernible in any model. We have also conducted this interaction effect analysis on the cut-off of HUA at the value of 5.6 mg/dL. Similarly, the additive interaction between HUA and HTN proved to be statistically significant. However, there was no significant interaction effect in multiplicative scale (**Supplementary Table S1**).

**Table 4:** Interaction effect indicators of HUA and HTN on CKD (n=1,756).

Models	Effect value			
	Additive scale			Multiplicative scale
	RERI(95%CI)	AP(95%CI)	SI(95%CI)	OR(95%CI)
Crude	2.26 (0.45-4.18)	0.42(0.09-0.60)	2.09(1.16-3.78)	1.48(0.68-3.22)
Model 1	2.24 (0.41-4.17)	0.43(0.08-0.61)	2.14(1.14-4.01)	1.47(0.68-3.22)
Model 2	2.57 (0.71-4.71)	0.47(0.14-0.64)	2.39(1.24-4.58)	1.65(0.74-3.67)

HUA, hyperuricemia; HTN, hypertension; OR, odds ratio; CI, confidence interval; RERI, relative excess risk due to interaction; AP, attributable proportion due to interaction; SI, synergy index.

Crude: unadjusted any covariate; Model 1: adjusted for age, gender; Model 2: adjusted for age, gender, educational level, FPG abnormal, HbA1c abnormal, TC abnormal, HDL-C abnormal and alcohol drinking, duration of diabetes.

## Discussion

Chronic kidney disease is a progressively worsening condition characterized by structural and functional disruptions within the renal system, arising from a range of etiologies (3). It is distinguished by its high prevalence and associated mortality rates, thus constituting a major global health concern (21).Our study is the pioneering investigation into the prevalence of CKD among patients with T2DM in Zhejiang Province, Eastern China. In 2018, this prevalence was found to be 27.62%, which is significantly higher than the general Chinese population’s rate of 8.2% (22), and Zhejiang province’s rate of 9.88% (23). One potential reason for this observation may be due to insulin resistance (IR) in T2DM patients. IR can cause vascular damage and is linked to various abnormal syndromes, including obesity, CKD and cardiovascular disease (CVD), etc. (24).Due to the substantial number of patients with T2DM in Zhejiang Province, this high prevalence of CKD poses a significant hidden risk to socio-economic development.

Serum uric acid is the end product of purine metabolism in the body and has important biological activities such as antioxidant under normal conditions (25). Our study revealed a U-shaped relationship between SUA levels and the risk of CKD, and this association remained significant after controlling for confounding factors. In brief, both low and high SUA levels were associated with the elevated risk of CKD. Previously published studies on other different populations had also found this U-shaped relationship. A 10-year prospective study by Kazuma Mori and colleagues found a U-shaped association of the baseline UA level with the risk of CKD in females (26). Eiichiro Kanda et al’s cohort study demonstrated that SUA level has a U-shaped association with loss of kidney function in healthy people (27). Yuta Matsukuma et al. revealed a J-shaped association between SUA levels and poor renal survival in female patients with IgA nephropathy (28).The mechanism linking low SUA levels to an increased risk of CKD is not that clear. One possible mechanism is that SUA’s antioxidant properties facilitate endothelial function preservation. Conversely, hypouricemia can induce renal artery spasms leading to Exercise-Induced Acute Kidney Injury (EIAKI). Past research has concluded that approximately 24% of EIAKI patients experience recurrent Acute Kidney Injury (AKI) cases (29). Despite exhibiting normal creatinine clearance rates, pathology findings revealed chronic conditions such as tubular basement membrane thickening and interstitial fibrosis in recurrent AKI patients (30). It’s suggested that the potential of recurrent AKI progressing into CKD (31). Besides, due to SUA’ antioxidant effect, a decrease in circulating SUA levels may reduce tissue tolerance to oxidative stress-mediated injury. Additionally, a low serum SUA level has been suggested to decrease eGFR by constricting afferent arterioles in the kidney (32).However, the role of elevated SUA in the progression of CKD is still debated. Some studies have shown that the elevated level of SUA is a risk factor for CKD (33–35). The possible mechanisms may be as follows: traditionally, it is believed that kidney damage due to HUA is the result of UA crystal impacts (36). Hypotheses exist that link acute renal damage to HUA, associated with hyperuricosuria, through the intraluminal crystal deposition in collecting ducts, initiating tubular obstruction and instigating inflammatory responses, leading to eventual tubulointerstitial harm (30). Moreover, other crystal-independent mechanisms have been proposed, including renal vasoconstriction mediated by endothelial dysfunction and activation of the renin-angiotensin system, etc. (30). On the other hand, several studies have indicated that treating HUA in patients with existing CKD does not improve kidney outcomes. Some experts even contend that it is unnecessary to treat asymptomatic HUA in CKD and suggest that HUA might actually be beneficial (37, 38). Consider the complexity of SUA’s role in CKD pathogenesis, the further investigations into the exact underlying mechanisms are needed.

In the conducted regression analyses, commonly recognized risk factors such as HUA and HTN displayed an association with a higher risk of CKD. The relationship between blood pressure (BP) and CKD is intricate, as HTN can either be an outcome of, or contribute to, a decline in kidney function (39). Several extensive prospective studies conducted on general populations and patients undergoing HTN treatment have indicated that BP operates as an autonomous risk factor for adverse renal outcomes. In other words, the risk of CKD and end-stage renal disease (ESRD) amplifies with increasing BP levels (40–44). Similarly, previous research has corroborated the role of HUA in exacerbating the deterioration of renal function (45, 46).In accordance with the listed prior researches, our study also revealed the independent association of HUA and HTN as risk factors in renal function decline.

Interactions are classified into multiplicative interactions and additive interactions. Interaction on the multiplicative scale shows whether the risk of an outcome from the combination of two risk factors significantly differs from the product of the individual risks (9). Interaction on the additive scale evaluates the difference between the event risk when both risk factors are present and the sum of the risks when only one risk factor is present (47). Therefore, it’s possible that only one type of interaction effect may be significant. Currently, there is no consensus on which scale is superior (9). However, the additive scale may hold greater clinical significance because it directly assesses the proportion of risk attributed to the synergistic effect of the two risk factors, unlike the multiplicative scale (9).The newly introduced STROBE statement advises authors to employ both multiplicative and additive scales of interaction when examining the combined effect of two risk factors (48). Consequently, we presented the interaction outcomes on both scales, with the aim of facilitating an easier comparison between our conclusions and those from comparable studies. Our analysis of the additive interaction revealed a synergistic joint effect of HUA and HTN on CKD in patients with T2DM. Meaning, the combined presence of HUA and HTN in T2DM patients has a more profound detrimental impact on CKD than their independent effects combined. Importantly, this indicates that the coexistence of HUA and HTN not only augments the impact but also precipitates greater harm. Based on our findings, patients with T2DM should be more aware of maintaining their healthy SUA and blood pressure levels. Our findings are noteworthy given the lack of comparable studies exploring this interaction in T2DM patients. Our results echo those of a preceding study concerning this interaction effect (9). However, this prior study, which only scrutinized the influence of HUA and HTN on the decline of e-GFR, was conducted on the general population. Therefore, our study serves to broaden the scope of the existing knowledge on this particular interaction.

Our study’s results have notable implications for both CKD prevention and management in individuals with T2DM. First, our study underscores the importance of routine screening for HUA and HTN in patients with T2DM. Clinicians should consider incorporating regular assessments of SUA and BP measurements into standard care protocols for T2DM patients. Early identification of elevated SUA and HTN can prompt timely interventions to mitigate the risk of CKD development. Second, the significant synergistic interaction between HUA and HTN suggests that an integrated approach to managing both conditions may be more effective in preventing CKD. Clinicians should develop comprehensive treatment plans that address both HUA and HTN simultaneously. This could involve the use of medications such as urate-lowering agents and antihypertensive drugs, along with lifestyle modifications including dietary changes, increased physical activity, and weight management. Furthermore, our research could serve as a foundation for developing public health recommendations and implementing more specific health intervention strategies.

## Limitation

Recognizing our study’s limitations is crucial. First, the cross-sectional design inhibits our capacity to determine causation between HUA, HTN and CKD among T2DM patients. Second, the potential for information bias emerges due to participants’ self-reporting of current cigarette smoking and alcohol drinking. Third, it is important to note that our research concentrated on a specific diabetic population in Eastern China, thus limiting the generalizability of our findings to other populations. Fourth, hypertension medications, especially diuretics, can affect SUA values. However, due to the lack of detailed information on hypertension medication treatments in the questionnaire, we were unable to include this in the analysis. Finally, although we identified most major confounders, there may still be residual confounding factors that could influence the study results. To substantiate our findings, future longitudinal studies should be more comprehensive and inclusive of larger, diverse cohorts.

## Conclusion

Our study is the first study to revealed a significant synergistic interaction between HUA and HTN in association with CKD among patients with T2DM. It also revealed a U-shaped relationship between SUA levels and the risk of CKD. This finding highlights the critical need to screen for and manage both HUA and HTN in patients with T2DM. The clinical implications for CKD prevention are substantial, suggesting that interventions targeting both conditions may be more effective than addressing either one in isolation. Additionally, understanding this complex relationship can inform future research focused on elucidating the biological pathways through which these conditions jointly contribute to renal impairment. These insights may lead to the development of therapeutic strategies that more effectively mitigate CKD progression in this high-risk population, ultimately shaping public health policies and improving patient outcomes.

## Data availability statement

The raw data supporting the conclusions of this article will be made available by the authors, without undue reservation.

## Ethics statement

The studies involving humans were approved by Shanghai Jiao Tong University Affiliated Sixth People’s Hospital. The studies were conducted in accordance with the local legislation and institutional requirements. The participants provided their written informed consent to participate in this study.

## Author contributions

X-yC: Methodology, Software, Writing – original draft, Writing – review & editing. FL: Investigation, Writing – review & editing. JZ: Investigation, Writing – review & editing. C-xX: Data curation, Writing – review & editing. X-fD: Data curation, Writing – review & editing. M-bL: Data curation, Writing – review & editing. L-jC: Data curation, Writing – review & editing. J-mZ: Conceptualization, Supervision, Writing – review & editing.
