# Supplementary material for: The effect of hyperuricemia and its interaction with hypertension towards chronic kidney disease in patients with type 2 diabetes: evidence from a cross- sectional study in Eastern China
Source: Front Endocrinol (Lausanne). 2024 Jul 29;15:1415459. doi: 10.3389/fendo.2024.1415459 (PMC11317236; doi:10.3389/fendo.2024.1415459)
Supplement: Supplementary file 1 [file DataSheet_1.doc]

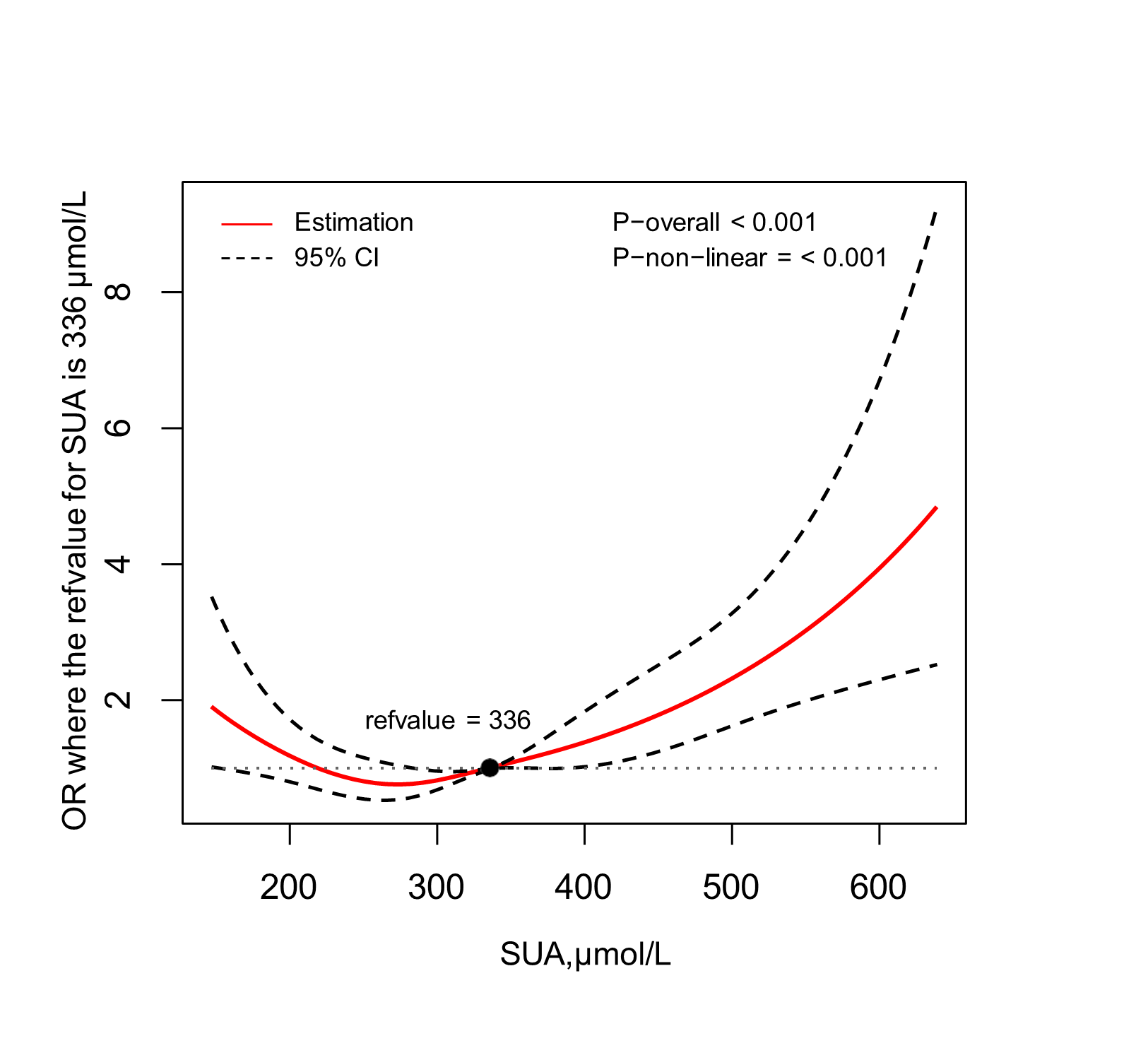


Figure caption:

Supplementary Figure S1. Association between SUA and the risk of CKD, allowing for nonlinear effects, with 95%CI. The model shows ORs compared with SUA=336μmol/L,adjusting for age,gender,educational level,HTN, FPG abnormal, HbA1c abnormal, TC abnormal, HDL-C abnormal, alcohol drinking and duration of diabetes. SUA, serum uric acid; CKD, chronic kidney disease;CI, confidence interval; OR, odds ratio; HTN,hypertension;FPG, fasting plasma glucose; TC,total cholesterol; HDL-C, high density lipoprotein-cholesterol.
