# Supplementary material for: The effect of hyperuricemia and its interaction with hypertension towards chronic kidney disease in patients with type 2 diabetes: evidence from a cross- sectional study in Eastern China
Source: Front Endocrinol (Lausanne). 2024 Jul 29;15:1415459. doi: 10.3389/fendo.2024.1415459 (PMC11317236; doi:10.3389/fendo.2024.1415459)
Supplement: Supplementary file 2 [file Table_1.docx]

**Supplementary information for**

The effect of hyperuricemia and its interaction with hypertension towards chronic kidney disease in patients with type 2 diabetes: evidence from a cross- sectional study in Eastern China

Xiang-yu Chen, Feng Lu, Jie Zhang, Chun-xiao Xu, Xiao-fu Du, Ming-bin Liang, Li-jin Chen, Jie-ming Zhong*

Department of Non-Communicable Disease Control and Prevention, Zhejiang Provincial Center for Disease Control and Prevention, Hangzhou, China

* Correspondence:

Jie-ming Zhong

jmzhong@cdc.zj.cn

**Supplementary Table S1. Interaction effect indicators of HUA and HTN on CKD (n=1,756)**

| Models | Effect value | | | |
| --- | --- | --- | --- | --- |
|  | Additive scale | | | Multiplicative  scale |
|  | RERI(95%CI) | AP(95%CI) | SI(95%CI) | OR(95%CI) |
| Crude | 1.52 (0.54-2.60) | 0.37(0.14-0.54) | 1.97(1.17-3.31) | 1.51(0.89-2.56) |
| Model 1 | 1.52 (0.54-2.63) | 0.38(0.14-0.55) | 2.01(1.17-3.45) | 1.48(0.87-2.52) |
| Model 2 | 1.68 (0.61-2.94) | 0.39(0.15-0.56) | 2.05(1.19-3.50) | 1.45(0.84-2.49) |

Abbreviations: HUA,hyperuricemia; HTN,hypertension; OR, odds ratio; CI, confidence interval; RERI, relative excess risk due to interaction; AP, attributable proportion due to interaction; SI, synergy index.

Crude: unadjusted any covariate; Model 1: adjusted for age,gender; Model 2: adjusted for age,gender,educational level, FPG abnormal, HbA1c abnormal, TC abnormal, HDL-C abnormal and alcohol drinking, duration of diabetes.
